# The importance of uterosacral ligament anatomy in overactive bladder: A preliminary study

**DOI:** 10.4274/tjod.73669

**Published:** 2018-03-29

**Authors:** Cevdet Adıgüzel, Esra Selver Saygılı Yılmaz, Sefa Arlıer, Sevtap Seyfettinoğlu, Gökhan Söker, Gülsüm Uysal, Oğuz Yücel, Akın Sivaslıoğlu

**Affiliations:** 1 Adana Numune Training and Research Hospital, Clinic of Obstetrics and Gynecology, Adana, Turkey; 2 Adana Numune Training and Research Hospital, Clinic of Radiology, Adana, Turkey; 3 Muğla Sıtkı Koçman University Faculty of Medicine, Department of Obstetrics and Gynecology, Muğla, Turkey

**Keywords:** Integral theory, magnetic resonance imaging, overactive bladder, uterosacral ligaments

## Abstract

**Objective:**

To evaluate whether uterosacral ligament (USL) thickness measured using magnetic resonance imaging (MRI) was associated with overactive bladder (OAB) in otherwise healthy women.

**Materials and Methods:**

The study comprised 27 women with OAB and 27 healthy women (control group) who were followed up at the Obstetrics and Gynecology Department of a tertiary referral center. All subjects were evaluated using pelvic MRI to determine the transverse USL thickness. These measurements were compared between the two groups. p values less than 0.05 were considered statistically significant.

**Results:**

The mean age of women in the OAB and control groups were 43.88±9.36 years and 39.92±5.36 years, respectively. The mean body mass index in the OAB group was 29.77±4.82 kg/m^2^ and 27.49±3.44 kg/m^2^ in the control group. In the comparison of Pelvic Organ Prolapse Quantification system stages between the groups, no statistically significant relationship was determined. In the OAB group, the mean right USL thickness was 2.04±0.34 mm, and the mean left USL was 2.04±0.52 mm. In the control group, the mean right USL thickness was 2.17±0.47 mm, and the mean left USL was 2.09±0.51 mm. There were no statistically significant differences in terms of USL thickness between the OAB and control groups (p>0.05).

**Conclusion:**

No previous studies have been identified in the literature that have investigated the relationship between USL thicknesses and urinary incontinence. In the present study, no significant relationship could be demonstrated between right and left USL thicknesses of the OAB and control groups. This was a preliminary study, and further research with larger sample sizes is required to reach a final conclusion.

**PRECIS:** No difference was identified in terms of right and left USL thicknesses of the OAB and control groups. This was a preliminary study, and further research with larger sample sizes is required to reach a conclusion.

## Introduction

Overactive bladder (OAB) is a significant health problem that can negatively affect quality of life^(1)^. “OAB” is a term that describes a syndrome of urinary urgency with or without incontinence, which is often accompanied by nocturia and urinary frequency^(2,3)^. The terms “urgency incontinence” and “OAB with incontinence” are often used interchangeably. Significant risk factors for urinary incontinence are primarily known as age, obesity, births, menopause, hysterectomy, and cigarette smoking^(4)^. It is thought to result from detrusor overactivity, leading to uninhibited detrusor muscle contractions during bladder filling^(3)^. In the etiology, neurologic disorders (e.g., spinal cord injury), bladder abnormalities, increased or altered bladder microbiome may be other reasons, or this may be idiopathic^(5)^. The integral theory describes the pathophysiology of urinary incontinence. The integral theory indicates that pelvic organ prolapses and abnormal pelvic symptoms such as urge, frequency, nocturia, and pelvic pain are usually caused by connective tissue laxity in the vagina or its supporting ligaments^(6)^. In the theory, the pelvic floor muscle forces the vaginal membrane to stretch against the suspensory ligaments to stimulate the micturition stretch receptors. Laxity in the membrane or suspensory ligaments may activate stretch receptors, which are perceived by the cortex as urgency, frequency, and nocturia untimely. The cortex then perceives this stimulation as urgency, frequency, and nocturia^(7)^. Imaging methods are playing an increasingly important part in the diagnosis of pelvic floor disorders. In many studies, pelvic floor disorders have been evaluated with magnetic resonance imaging (MRI)^(8)^. Measurements of the uterosacral ligaments (USL), which are strong ligaments of the uterus, have previously been made with ultrasound in cadaver studies and in patienst with endometriosis^(9,10,11)^. The aim of the current study was to investigate the role of USL anatomy in stretching the vaginal membrane in patients with OAB.

## Materials and Methods

**Figure 1:**
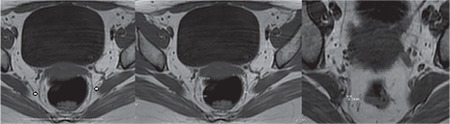
Measurement points of the uterosacral ligament on magnetic resonance imaging

Approval for this controlled clinical study was granted by the Adana Numune Training and Research Hospital Ethics Committee (approval number: 122/03.11.2015) and written informed consent was obtained from all participants. The study included a total of 27 patients who had been diagnosed as having OAB in our clinic between January 2013 and December 2015. Patients were excluded from the study if they were determined to have concomitant stress urinary incontinence or a malignant pathology. The control group comprised 27 healthy women. The diagnosis of OAB was made from the anamnesis and physical examination. On the first presentation, the patients were questioned in respect of the times that urine leakage occurred, the amount of urine leakage, the reason for the leakage, and what increased or decreased the leakage. Questions were also asked regarding the presence of additional diseases that could cause urine leakage. A physical and pelvic examination was performed to all patients. The stress test was applied. The patients kept a urine diary and this was examined. Patients with OAB who met the study criteria were included in the evaluation. For all the patients included in the study, a record was made of age, height, weight, body mass index (BMI), parity, type of births, and the Pelvic Organ Prolapse Quantification system (POP-Q) was used, which was first published in 1996, in an article by Bump et al.^(12)^ The hymen acts as the set point of indication throughout the POP-Q staging. There are six described points for quantity in the POP-Q system. Anterior: Aa, Ba, C, posterior: Ap, Bp, D. Three others milestones: genital hiatus, the vaginal length (TVL), and perineal body. Each is measured in centimeters above or proximal to the hymen (negative number) or centimeters below or distal to the hymen (positive number) with the plane of the hymen being defined as zero (0). Stage 0: no prolapse; Stage I: the most distal portion of prolapse is >1 cm above level of hymen; Stage II: the most distal part of prolapse is <1 cm proximal to or distal to the plane of hymen; Stage III: the most distal portion of the prolapse protrudes more than 1 cm below the hymen but no farther than 2 cm less than the total TVL (for example, not all of the vagina has prolapsed); Stage IV: complete vaginal eversion is needed, full urine test and urine culture. Lower abdominal MRI was taken using a 1.5 Tesla MRI system (Siemens Magnetom Avanto, Philadelphia, USA). The same protocol was applied to all patients and the control group. Axial T1 and T2 sequences and coronal and sagittal T2 sequences were used. The parameters for the sequences used in the study were as follows: T1-weighted axial repetition time (TR): 370 ms, echo time (TE): 7.11 ms, slice thickness: 3 mm, field of view (FOV): 34x34 mm; T2-weighted axial TR: 3185 ms, TE: 105 ms, slice thickness: 3 mm, FOV: 33x33 mm; T2-weighted turbo spin echo (TSE) sagittal TR: 5117 ms, TE: 120 ms, slice thickness: 3 mm, FOV: 30x30 mm; T2-weighted TSE coronal TR: 5484 ms, TE: 120 ms, slice thickness: 3 mm, FOV: 32x32 mm. The images were evaluated on a separate workstation by a radiology specialist with 10 years’ experience who was blinded to the study. All images were investigated in respect of pelvic pathology, then the USLs on both sides were identified and an evaluation was made concerning their thickness and nodularity. The USL thickness was measured using MRI at the closest points to the cervix and the sacrum and at the mid point between those two points, and the results of the OAB group were compared with those of a control group. Any patients with nodularity that was found to be significant for endometriosis were excluded from the study. A measurement was made of the transverse thickness of the area observed as hypointense on T1 and T2-weighted sequences from the closest points to the cervix and the sacrum and from the midpoint of those two points (Figure 1). The mean of the three measurements was then calculated for the right and left USL.

### Statistical Analysis

The statistical analysis of the study data was made using Statistical Package for Social Sciences (SPSS) v. Twenty-one software (SPSS , Chicago, IL, USA). Comparisons were made between the OAB patient group and the control group in respect of the above-mentioned clinical parameters and the USL thickness measured on MRI. Categorical variables were stated as number and percentage (%) and numerical variables as mean (minimum-maximum) ± standard deviation (SD). Conformity of the data to normal distribution was assessed using the the Kolmogorov-Smirnov test. In the comparisons of categorical data between the groups, the chi-square test or Fisher’s exact test was used. In the comparison of numerical data, Student’s t-test was used when there was conformity to normal distribution and the Mann-Whitney U test where there was non-normal distribution. A value of p<0.05 was accepted as statistically significant.

## Results

The study included a total of 33 patients with OAB and 30 control subjects who met the study criteria within the specified period. A total of 6 patients with OAB and 3 control group subjects were excluded from the final evaluation because the MRIs were not clear. The mean age was 43.88±9.36 years in the OAB group and 39.92±5.36 years in the control group. The mean BMI value was 29.77±4.82 kg/m^2^ in the OAB group and 27.49±3.44 kg/m^2^ in the control group. The mean parity was 3.37±1.59 in the OAB group and 2.7±1.68 in the control group (Table 1). In the OAB group, previous births were performed with vaginal delivery in 59.3%, caesarean section in 25.9%, and with vaginal delivery and cesarean in 14.8%. In the control group, previous births were performed with vaginal delivery in 55.6%, with cesarean section in 37%, and with vaginal delivey and cesarean in 7.4% (p>0.05) (Table 2). No statistically significant difference was determined between the groups in respect of episiotomy (p=0.08). In the comparison of the POP-Q stages between the groups, no statistically significant relationship was determined (p>0.05) (Table 3). The mean (± SD) thickness of the right USL and left USL of both the OAB and control groups was 2.10±0.4 mm and 2.06±0.51 mm, respectively. In the OAB group, the mean thickness of the right USL was 2.04±0.34 mm and the mean thickness of the left USL was 2.04±0.52 mm. In the control group, the mean thickness the right USL was 2.17±0.47 mm and the mean thickness of the left USL was 2.09±0.51 mm. No statistically significant difference was found between the groups in respect of the thickness of the right USL (p=0.71) and the thickness of the left USL (p=0.206) (Table 4).

## Discussion

In current study, we aimed to evaluate thickness of the USL using MRI to find anatomic disorders of USL that may cause laxness of the vaginal membrane. We hypothesized that the thickness of the USL might correlate with OAB. The role of imaging methods in the evaluation of pelvic floor dysfunctions has been questioned in many studies. MRI in particular has become increasingly useful in the diagnosis of pelvic organ prolapse and pelvic floor disorders^(12,13)^. In previous studies, a correlation has been determined between pelvic floor measurements made with MRI and POP clinical staging^(14)^. However, as study data are limited and cannot be compared, there is no standardized method as yet for MRI measurements^(15)^. In a study by Tan et al.^(8)^ pelvic MRI was performed in young healthy females and cadavers, clearly showing the pelvic and urogenital diaphragm and the previously defined uterus-supporting tissues, and the periuretal and parauretal ligaments were able to be seen anatomically. Stoker et al.^(16)^ examined the whole pelvic floor aiming to find a solution to both urinary and anal dysfunction. The integral theory of female urinary incontinence states that stress and urge symptoms both derive from the same anatomical defect, a lax vagina^(17)^. According to the integral theory, urge, frequency, and nocturia are neurogenic symptoms and can happen with minimal prolapse^(6)^. The integral theory suggests sustentation of the mid-urethra (the anterior vaginal wall along the arcus tendineous, and the vaginal cuff along the uterosacral “neoligament”) will prevent a lax vaginal membrane, which will cure OAB and/or urge incontinence symptoms. This is based on the presence of hypothesized stretch receptors at the proximal urethra and bladder neck^(6)^. On the other hand, hysterectomy has been implicated as a risk factor for the development of urinary incontinence^(18)^. Urinary incontinence after hysterectomy can be the result of a lasting injury to the pelvic plexus at the time of uterosacral/cardinal ligament complex transection, bladder flap formation, and possibly disruption of the anatomic support to the bladder neck and urethra^(19)^. The USL is an important ligament that supports the pelvic floor. No systematic mapping has been performed to define the length and thickness of the USL^(10)^ and there are very few studies in the literature with any information related to USL thickness. In a cadaver dissection study by Vu et al.^(9)^, the USL thickness was reported to be a mean 5-20 mm in the cervical region, 5 mm in the central region, and 5 mm in the sacral region. In addition to cadaver studies, USL thickness has been evaluated in endometriosis. In a study by Bazot et al.^(20)^ MRI findings showed USL thickness as >9 mm in patients with endometriosis. In our study, we were also able to identify the POP-Q stages between the control and OAB groups. However, a statistically significant relationship was not determined between the two groups. It is possible that laxness of the USL may be correlated with both thickness of the ligament and the distribution and function of the collagen types within the tissue^(21,22,23)^. In the whole group of the current study, including the OAB patients and the control group, the mean thickness of the left USL was 2.06±0.51 mm, and the right was 2.10±0.40 mm. None of the current study patients had endometriosis or any pelvic pain. In the OAB group, the mean thickness of the right USL was determined as 2.04±0.34 mm, and the left as 2.04±0.52 mm. In the control group, the mean thickness of the right USL was 2.17±0.46 mm, and the left was 2.09±0.51 mm (Table 4). According to the integral theory, vagina and bladder base defects are displayed as “OAB” symptoms. A previous study by Petros and Ulmsten^(7)^ reported that repair of ligament stretch and tension restored anatomy and function. The USLs are major insertion points for the directional vectors that stretch the vaginal membrane to block premature activation of the micturition reflex. All different appearances of a prematurely activated micturition reflex such as urge incontinence depend on the link between loose ligaments and diminished striated muscle force. The Testicular Feminisation syndrome creates a strong suspension structure to restore muscle contractility and to prevent urge incontinence, as well as urge symptoms^(6,23,24,25)^.

### Study Limitations

This study provides useful preliminary data of the relationship between USL thickness and incontinences. However, the present study has some limitations. First, the sample size was small. Due to the low number of patients, this report should be regarded as a preliminary study. A larger number of patients may be more likely to elucidate the relationship of USL thickness with OAB and the development of urinary problems. Second, a urodynamic test was not used to determine causes of incontinence.

## Conclusion

In conclusion, no statistically significant difference was found between the two groups examined in respect of both left and right USL thickness. There is a need for further studies including a larger number of patients.

## Figures and Tables

**Table 1:**
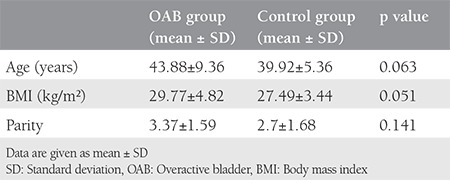
Demographic characteristics of overactive bladder and control groups

**Table 2:**
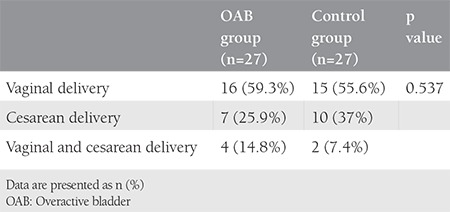
Obstetric history of overactive bladder and control groups

**Table 3:**
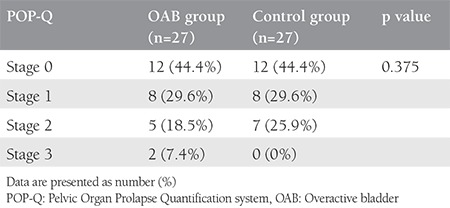
Pelvic Organ Prolapse Quantification system staging of overactive bladder and control groups

**Table 4:**
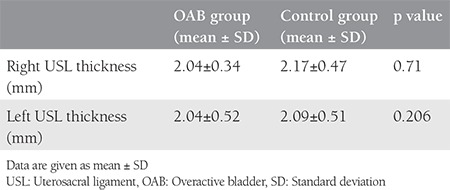
Uterosacral ligament thickness of overactive bladder and control groups
